# Reproducibility of methodological radiomics score (METRICS): an intra- and inter-rater reliability study endorsed by EuSoMII

**DOI:** 10.1007/s00330-025-11443-1

**Published:** 2025-02-19

**Authors:** Tugba Akinci D’Antonoli, Armando Ugo Cavallo, Burak Kocak, Alessandra Borgheresi, Andrea Ponsiglione, Arnaldo Stanzione, Emmanouil Koltsakis, Fabio Martino Doniselli, Federica Vernuccio, Lorenzo Ugga, Matthaios Triantafyllou, Merel Huisman, Michail E. Klontzas, Romina Trotta, Roberto Cannella, Salvatore Claudio Fanni, Renato Cuocolo

**Affiliations:** 1 Institute of Radiology and Nuclear Medicine, Cantonal Hospital Baselland, Liestal, Switzerland; 2 Division of Radiology, Istituto Dermopatico dell’Immacolata (IDI), IRCCS, Rome, Italy; 3 Department of Radiology, Basaksehir Cam and Sakura City Hospital, University of Health Sciences, Istanbul, Turkey; 4 Department of Clinical, Special and Dental Sciences, University Politecnica delle Marche, Ancona, Italy; 5 Department of Radiology, University Hospital “Azienda Ospedaliero Universitaria delle Marche”, Ancona, Italy; 6 Department of Advanced Biomedical Sciences, University of Naples Federico II, Naples, Italy; 7 Department of Radiology, Karolinska University Hospital of Stockholm, Stockholm, Sweden; 8 Neuroradiology Unit, Fondazione IRCCS Istituto Neurologico Carlo Besta, Milano, Italy; 9 Department of Biomedicine, Neuroscience, and Advanced Diagnostics (Bi.N.D.), University of Palermo, Palermo, Italy; 10 Department of Medical Imaging, University Hospital of Heraklion, Crete, Greece; 11 Department of Radiology and Nuclear Medicine, Radboud University Medical Center, Nijmegen, The Netherlands; 12 Artificial Intelligence and Translational Imaging (ATI) Lab, Department of Radiology, School of Medicine, University of Crete, Heraklion, Greece; 13 Computational Biomedicine Lab, Institute of Computer Science, Foundation for Research and Technology (ICS-FORTH), Crete, Greece; 14 Division of Radiology, Department of Clinical Science, Intervention and Technology (CLINTEC), Karolinska Institute, Stockholm, Sweden; 15 Department of Radiology, Santa Clotilde’s Hospital, Santander, Spain; 16 Department of Translational Research, Academic Radiology, University of Pisa, Pisa, Italy; 17 Department of Medicine, Surgery, and Dentistry, University of Salerno, Fisciano, Italy

**Keywords:** Artificial intelligence, Radiomics, Reproducibility of results, Inter-observer variability, Intra-observer variability

## Abstract

**Objectives:**

To investigate the intra- and inter-rater reliability of the total methodological radiomics score (METRICS) and its items through a multi-reader analysis.

**Materials and methods:**

A total of 12 raters with different backgrounds and experience levels were recruited for the study. Based on their level of expertise, raters were randomly assigned to the following groups: two inter-rater reliability groups, and two intra-rater reliability groups, where each group included one group with and one group without a preliminary training session on the use of METRICS. Inter-rater reliability groups assessed all 34 papers, while intra-rater reliability groups completed the assessment of 17 papers twice within 21 days each time, and a “wash out” period of 60 days in between.

**Results:**

Inter-rater reliability was poor to moderate between raters of group 1 (without training; ICC = 0.393; 95% CI = 0.115–0.630; *p* = 0.002), and between raters of group 2 (with training; ICC = 0.433; 95% CI = 0.127–0.671; *p* = 0.002). The intra-rater analysis was excellent for raters 9 and 12, good to excellent for raters 8 and 10, moderate to excellent for rater 7, and poor to good for rater 11.

**Conclusion:**

The intra-rater reliability of the METRICS score was relatively good, while the inter-rater reliability was relatively low. This highlights the need for further efforts to achieve a common understanding of METRICS items, as well as resources consisting of explanations, elaborations, and examples to improve reproducibility and enhance their usability and robustness.

**Key Points:**

***Questions*** *Guidelines and scoring tools are necessary to improve the quality of radiomics research; however, the application of these tools is challenging for less experienced raters*.

***Findings*** *Intra-rater reliability was high across all raters regardless of experience level or previous training, and inter-rater reliability was generally poor to moderate across raters*.

***Clinical relevance*** *Guidelines and scoring tools are necessary for proper reporting in radiomics research and for closing the gap between research and clinical implementation. There is a need for further resources offering explanations, elaborations, and examples to enhance the usability and robustness of these guidelines*.

## Introduction

Radiomics encompasses a collection of analysis techniques enabling the extraction of high-dimensional feature datasets from biomedical images [1]. Being a rapidly evolving and growing field of image analysis technique, an exponential number of radiomics-related articles have been published since its introduction into medicine [2]. However, a significant translational gap exists between radiomics research and clinical practice [3–5], at least in part related to the poor quality and limited standardization of research methodology [3, 6–8] that limits its reproducibility. The complexity of such advanced methods that are used to extract and analyze quantitative imaging features may also cause difficulties in understanding all aspects of the analysis and in evaluating the research quality. Although radiomics offers explainability by making use of hidden biomarkers in the images, the complexity, as well as limited reproducibility, hinder the implementation of these techniques in the clinical setting [4]. For these reasons, standardization efforts [9], as well as the development of tools to evaluate the quality of radiomics research, have been valuable.

In 2017, Lambin et al [10] proposed the radiomics quality score (RQS), a methodological assessment tool that breaks down the radiomics analytical process into several steps for evaluation. It comprises 16 items covering the entire lifecycle of radiomics research. The interpretation of items, and so the final score, suffers from subjective analysis which can be influenced by different factors, such as the experience of the reader in the interpretation of RQS or the overall experience in radiological practice. For example, the ambiguity in some RQS item definitions may limit their understandability and some items can be specific to one study design and cannot apply to another [11–14]. In addition, a high RQS score does not always guarantee high study quality [15]. In 2023, an RQS reproducibility study reported that inter-rater reliability was poor to moderate whereas intra-rater reliability was moderate to good, suggesting considerable subjectivity [13].

The methodological radiomics score (METRICS) [16] was recently proposed by a large international group of experts in the field of radiomics research and specifically aimed at improving the methodological quality of radiomics research and designed to be easy to use. The final METRICS tool includes 30 items (with relative item weights), organized within 9 categories (study design, imaging data, segmentation, imaging processing and feature extraction, feature processing, preparation for modeling, metrics and comparison, testing, and open science). It also accounts for different study pipelines by including several conditional items. METRICS was developed by following a modified Delphi protocol and endorsed by the European Society of Medical Imaging Informatics (EuSoMII) [16]. The final METRICS score is calculated by the aggregation of all individual weighted item scores and reported as a percentile along with the quality categories (https://metricsscore.github.io/metrics/METRICS.html). Key differences between the previous RQS and METRICS include their development processes and scope. METRICS was created through a rigorous, transparent modified Delphi process involving a large international expert group, whereas RQS was developed by a smaller group without transparency. Additionally, METRICS encompasses both handcrafted and deep radiomics workflows, addressing critical areas that RQS overlooks, such as “study design,” “segmentation,” “image processing and feature extraction,” and “preparation for modeling [17]. METRICS stands out for its user-friendliness, offering clear item definitions, conditional elements, and an intuitive web application for scoring, which minimizes potential errors or inappropriate scoring that was previously widely observed in the use of RQS [6].

This design makes METRICS highly preferable for radiomics researchers, journal reviewers, and editors who prioritize usability, transparency, reproducibility, and open science practices when evaluating radiomics studies. By facilitating comprehensive evaluations, METRICS helps ensure high-quality standards in radiomics research. It is also a valuable tool for systematic reviews, enabling the comparison of study quality across published radiomics research papers.

Given its novelty, the score has not been widely tested and, more importantly, no dedicated reproducibility study has been performed yet. We believe reproducibility is essential for ensuring the robustness of tools proposed to evaluate radiomics studies, as it helps reduce individual error and variability. Consequently, robust quality assessment of radiomics models is crucial to enhancing their potential for effective integration into clinical practice. The purpose of this study was to determine the intra- and inter-rater reliability of the total METRICS score and its individual items through a multi-reader study.

## Material and methods

### Paper selection

We included studies published in the first- and second-quartile (Q1 and Q2) radiology journals based on citation reports of Web of Science, within an arbitrarily chosen period of 01/01/24 and 17/01/24. We randomly selected this timeframe by going retrospectively from the day we initiated the study until the beginning of the new year.

The following search query was used on Pubmed database: ((((((“radiomics” [MeSH Terms] OR “radiomics” [All Fields] OR “radiomic” [All Fields]) NOT (“comment” [Publication Type] OR “commentary” [All Fields])) NOT (“systematic review” [Publication Type] OR “systematic reviews as topic” [MeSH Terms] OR “systematic review” [All Fields])) NOT (“review” [Publication Type] OR “review literature as topic” [MeSH Terms] OR “review” [All Fields])) NOT (“editorial” [Publication Type] OR “editorial” [All Fields])) NOT (“comment” [Publication Type] OR “editorial comment” [All Fields])) AND (2024:2024 [pdat]).

**Fig. 1:**
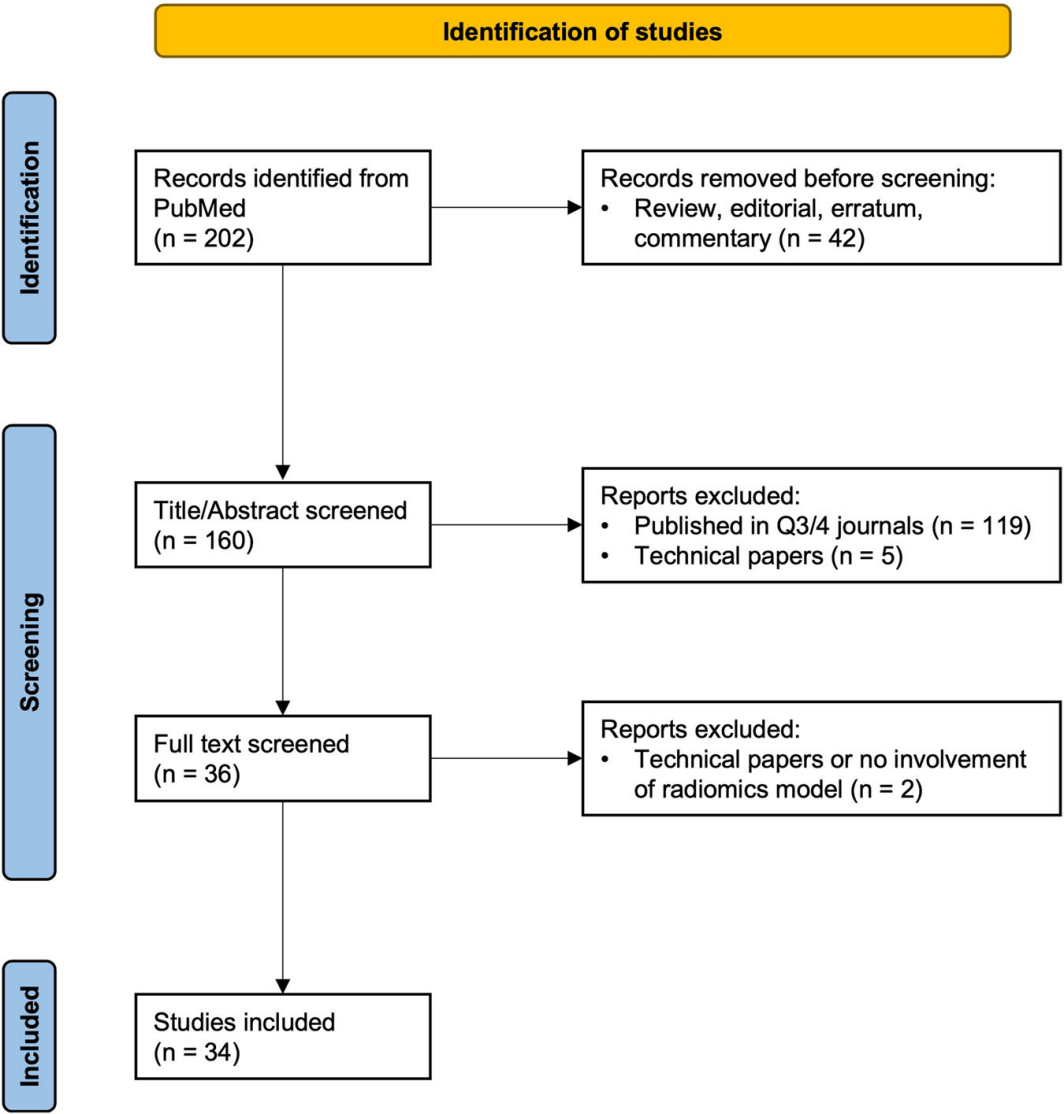
Identification of studies

Only original research articles were included, excluding systematic reviews, narrative literature reviews, editorials, letters, and corrections. After applying the exclusion criteria, a total of 34 articles were selected for the study, which was above the minimum suggested sample size, i.e., 30, for the inter-rater reliability studies based on the guideline of selecting and reporting intraclass correlation coefficients (ICC) for reliability research (Fig. 1) [18]. Table 1 shows the characteristics of the included studies.

**Table 1:** Characteristics of the included papers

Paper#	First author	Source title	Publication year	Country	Study design	Topic	Subtopic	Modality	Model Utility
1	Li Y	Eur Radiol Exp	2024	China	Retrospective	Brain	Metastases	MRI	Classification
2	Kelly BS	AJNR Am J Neuroradiol	2024	Ireland	Retrospective	Brain	Multiple sclerosis	MRI	Prognostication
3	Zeng X	Sci Rep	2024	China	Retrospective	Abdominal	Inflammatory bowel disease	CT	Prediction
4	Gao Y	BMC Cancer	2024	China	Retrospective	Head and neck	Thyroid	US	Classification
5	Wang P	Acad Radiol	2024	China	Retrospective	Chest	Lung cancer	CT	Prediction
6	Gross M	Eur Radiol	2024	USA	Retrospective	Abdominal	Liver	MRI	N/A
7	Yang X	BMC Cancer	2024	China	Retrospective	Neuro	Brain tumors	MRI, CT	N/A
8	Schön F	Sci Rep	2024	Germany	Retrospective	Abdominal	Hepatocellular carcinoma	CT	Prognostication
9	Jeong S	Sci Rep	2024	Korea	Retrospective	Genitourinary	Cervical cancer	MRI	Prognostication
10	Yu H	BMC Pulm Med	2024	China	Retrospective	Chest	Pneumocystis pneumonia	CT	Classification
11	Jia J	Acad Radiol	2024	China	Retrospective	Gastrointestinal	Esophageal cancer	CT	Prognostication
12	Deng Y	BMC Med Imaging	2024	China	Retrospective	Genitourinary	Kidney cancer	CT	Classification
13	Lin X	Eur Radiol	2024	China	Retrospective	Chest (cardiovascular)	COPD	CT	Prognostication
14	Huang L	Diagn Interv Radiol	2024	China	Retrospective	Genitourinary	Kidney lesions	CT	Classification
15	Kim S	Sci Rep	2024	Korea	Retrospective	Chest	Lung cancer	CT	Classification
16	Liu J	Eur Radiol Exp	2024	China	Retrospective	Chest	Pulmonary nodules	CT	Classification
17	Gong J	Cancer Imaging	2024	China	Retrospective	Brain	Brain metastasis	CT	Prognostication
18	Li F	Acad Radiol	2024	China	Retrospective	Breast	Breast cancer	US	Classification
19	Zhou L	Insights Imaging	2024	China	Retrospective	Chest	Lung cancer	18F FDG PETCT	Prognostication
20	Li Q	Insights Imaging	2024	China	Retrospective	MSK	Chondrosarcoma	CT	Prognostication
21	Liu Q	Eur Radiol	2024	China	Retrospective	Head and neck	Laryngeal cancer	CT	Classification
22	Lu J	Sci Rep	2024	China	Retrospective	Chest	Lung cancer	CT	Classification
23	Zhang X	Radiother Oncol	2024	China	Retrospective	Chest	Lung cancer	CT	Classification
24	Li J	BMC Cancer	2024	China	Retrospective	Chest	Radiation pneumonitis	CT	Prognostication
25	Ebrahimi B	J Cardiovasc Magn Reson	2024	USA	Prospective	Vascular	Renal artery stenosis	MRI	Prognostication
26	Tian W	Cancer Imaging	2024	China	Retrospective	Chest	Lung cancer	CT	Prognostication
27	Yang P	Phys Eng Sci Med	2024	China	Retrospective	Abdominal	Liver	CT	Prognostication
28	Ma C	Korean J Radiol	2024	China	Retrospective	Neurological	Brain	MR	Prognostication
29	Lee JH	Cancer Imaging	2024	Korea	Retrospective	Abdominal	Pancreatic cancer	CT	Prognostication
30	Zheng H	Cancer Med	2024	China	Retrospective	Breast	Breast cancer	MRI	Prognostication
31	Knuth F	Sci Rep	2024	Norway	Retrospective	Abdominal	Rectal cancer	MRI	Prognostication
32	Neher P	Nat Commun	2024	Germany	retrospective	Neuro	Neurological degenerative diseases	MRI	Classification
33	Xiao L	Abdom Radiol	2024	China	Retrospective	Abdominal	Liver fibrosis	MRI	Classification
34	Feng B	Acad Radiol	2024	China	Retrospective	Abdominal	Hepatocellular carcinoma	MRI	Classification

*MRI* magnetic resonance imaging, *CT* computed tomography, *US* ultrasound, *PET* positron emission tomography, *N/A* not applicable

### Rater selection and raters’ survey

A total of 12 raters with different backgrounds and experience levels were recruited for the study with an open call within the EuSoMII Radiomics Auditing Group (Table 2). All raters first completed an initial survey, which was sent by email, with questions aimed at determining their level of expertise in radiomics research and quality score application, as well as the level of expertise in their occupation, all of which contributed to defining raters’ expertise level for the current study (i.e., exp1 = beginner, exp2 = intermediate, exp3 = advanced). Based on their level of expertise, they were later randomly assigned to the following groups: 2 inter-rater reliability groups, and 2 intra-rater reliability groups, with each pair of groups comprising one group with and one group without a preliminary training session on the use of METRICS.

**Table 2:** Characteristics of group participants

Rater	Raters’ number	Radiomics research	Applying RQS/TRIPOD	Total experience score	Years of experience	Occupation	Group
R.T.	1	1. None	1. None	2 (exp1)	11	Radiologist	1
F.M.D.	2	2. Less than ten original papers	3. Equal or more than ten papers	5 (exp2/years of experience)	9	Radiologist	1
A.P.	3	2. Less than ten original papers	3. Equal or more than ten papers	5 (exp3/years of experience)	10	Radiologist	1
E.K.	4	2. Less than ten original papers	1. None	3 (exp1)	4	Radiology resident	2
R.C.	5	3. Equal or more than ten original papers	2. Less than ten papers	5 (exp2)	9	Radiologist	2
A.S.	6	3. Equal or more than ten original papers	3. Equal or more than ten papers	6 (exp3)	10	Radiologist	2
M.T.	7	2. Less than ten original papers	2. Less than ten papers	4 (exp1)	2	Radiology resident	3
F.V.	8	2. Less than ten original papers	3. Equal or more than ten papers	5 (exp2)	10	Radiologist	3
L.U.	9	3. Equal or more than ten original papers	3. Equal or more than ten papers	6 (exp3)	11	Radiologist	3
A.B.	10	2. Less than ten original papers	2. Less than ten papers	4 (exp1)	10	Radiologist	4
S.C.F.	11	2. Less than ten original papers	3. Equal or more than ten papers	5 (exp2)	5	Radiologist	4
M.K.	12	3. Equal or more than ten original papers	3. Equal or more than ten papers	6 (exp3)	11	Radiology resident	4

*RQS* radiomics quality score, *TRIPOD* transparent reporting of a multivariable prediction model for individual prognosis or diagnosis, *exp* experience

The inter-rater reliability group with training (group 2) and the intra-rater reliability group with training (group 4) received an hour-long training session for explanation and demonstration of the METRICS assessment, where raters received 3 random articles (not part of the final study set) a week before the training session [19–21]. The raters independently assessed the training papers within that dedicated week beforehand, and during the training session, the raters were instructed by the METRICS steering committee (T.A.D., R.C., and B.K.) regarding the correct interpretation of all items on the same papers. The training session also included an open discussion to clarify any additional questions on METRICS item interpretation by the readers.

**Fig. 2:**
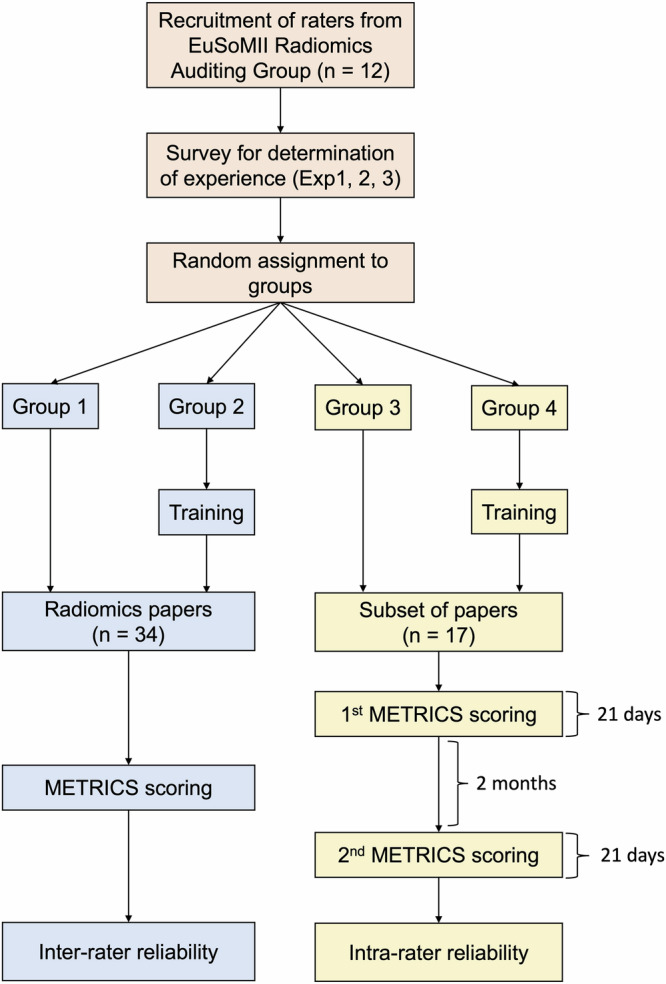
Study design. The subset of 17 papers was randomly selected among the initial 34 papers for intra-rater analysis. EuSoMII, the European Society of Medical Imaging Informatics; Exp, experience

Inter-rater reliability groups assessed all 34 papers [22–55], while intra-rater reliability groups assessed 17 randomly selected papers among the original 34 papers [22–38] twice within 21 days, with a 60-day washout period between assessments. All raters provided their ratings as they read the article, which is available in Supplementary Material. A keyword search was also allowed if needed. The overall study design is summarized in Fig. 2.

#### Post-scoring survey

To measure the usability of the METRICS tool and gather feedback from the raters we have also conducted a post-scoring survey, which included the same question about each item’s understandability and usability with responses categorized into three different levels (very clear and easy-to-use, a little challenging to understand and use, very difficult to understand and use).

### Statistical analysis

The ICC (two-way, single rater, agreement, random effects model for inter-rater analysis; two-way, single rater, consistency, and mixed effects model for intra-rater analysis [18]) was used for continuous variables, i.e., total METRICS score. ICC values were interpreted with the following scale: 0–0.5: poor, 0.5–0.75: moderate, 0.75–0.9: good, > 0.9: excellent [18]. Unless otherwise specified, 95% confidence interval values were considered in the interpretation of reliability as recommended for ICC [18].

Fleiss’ and Cohen’s kappa (k) statistics were used for categorical variables, i.e., item scores, as recommended [18, 56, 57]. Fleiss’ k was used to assess agreement when there were more than two raters/ratings [58], while Cohen’s k was when there were two ratings/raters. k values were interpreted as follows: ≤ 0: no agreement, 0.01–0.20: none to slight, 0.21–0.40: fair, 0.41– 0.60: moderate, 0.61–0.80: substantial, and 0.81–1.00: almost perfect agreement [58].

We used two one-sided *t*-tests (TOST), a test of equivalence based on the classical *t*-test, to investigate equivalence and differences between mean METRICS scores amongst rater groups [59]. To identify papers with the highest difference in total METRICS scores between raters in inter-rater analysis (group 1 [without training] and group 2 [with training]), the absolute pairwise differences between total METRICS scores calculated by single raters were calculated and then averaged.

The mean of the absolute difference between total METRICS scores was calculated to investigate which papers had very poor reproducibility in inter-rater analysis.

A *p*-value of 0.05 was used to determine statistical significance. All statistical analyses were conducted using R software (version 4.1.1), with the “irr” and “TOSTER” packages [R Core Team (2021). R: A language and environment for statistical computing. https://www.r-project.org/].

## Results

### Inter‑rater reliability

The inter-rater reliability was poor to moderate between raters of group 1 (without training; ICC = 0.393; 95% CI = 0.115–0.630; *p* = 0.002), as well as between raters of group 2 (with training; ICC = 0.433; 95% CI = 0.127–0.671; *p* = 0.002).

Regarding the inter-group analysis (i.e., without training vs with training), the reliability was poor to moderate between rater 1 vs 4 (ICC = 0.376; 95% CI = −0.081–0.684; *p* = 0.063) and between rater 1 vs 6 (ICC = 0.524; 95% CI = 0.234–0.729; *p* = 0.001), and moderate to excellent in the rater 3 vs 6 assessment (ICC = 0.869; 95% CI = 0.737–0.935; *p* < 0.001). Additionally, reliability based on ICC estimates was found to range from poor to good when comparing the ratings of groups 1 (without training) and 2 (with training) with the same level of experience (ICC = 0.376–0.869).

For intra-group analysis (i.e., both without training or both with training), the reliability based on ICC estimates was poor to moderate in both groups.

Detailed inter-rater analysis results for the total METRICS score are presented in Table 3 and Supplementary Material S1.

**Table 3:** Inter-rater reliability analysis for total METRICS score

Analysis	Target	ICC	95% CI	*p*
“Overall” comparison	Group 1	0.393	0.115–0.630	0.002
	Group 2	0.433	0.127–0.671	0.002
Within group comparison	Group 1			
	1 vs 2	0.351	−0.099 to 0.674	0.084
	1 vs 3	0.556	0.269–0.751	< 0.001
	2 vs 3	0.308	−0.071 to 0.608	0.063
	Group 2			
	4 vs 5	0.464	0.064–0.716	0.012
	4 vs 6	0.300	−0.093 to 0.615	0.080
	5 vs 6	0.604	0.210–0.806	0.002
Between-group comparison	1 vs 4	0.376	−0.081 to 0.684	0.063
	1 vs 5	0.612	0.328–0.791	< 0.001
	1 vs 6	0.524	0.234–0.729	0.001
	2 vs 5	0.343	−0.012 to 0.618	0.030
	2 vs 6	0.320	−0.104 to 0.655	0.104
	3 vs 6	0.869	0.737–0.935	< 0.001
Inter-group comparison (matched level of experience)	1 vs 3	0.376	−0.081 to 0.684	0.063
	2 vs 5	0.343	−0.012 to 0.618	0.030
	3 vs 6	0.869	0.737–0.935	< 0.001

“Overall” comparison refers to the comparison of estimate and CIs of ICC coefficients by all raters of the groups

Group 1: Inter-rater group without training (raters 1–3)

Group 2: Inter-rater group with training (raters 4–6)

Between-group comparison shows the comparison between two groups w and w/o training

*ICC* intra-class correlation, *CI* confidence interval

The reproducibility of quality categories was none-to-fair for all combinations, except for expert rates comparison (raters 3 vs 6), where it was moderate, as shown in Table 4.

**Table 4:** Inter-rater reliability analysis for METRICS quality categories

Analysis	Target	k (Fleiss)	*p*
“Overall” comparison	Group 1	−0.013	0.848
	Group 2	0.160	0.017
Within group comparison	Group 1	k (Cohen)	*p*
	1 vs 2	0.021	0.833
	1 vs 3	−0.098	0.390
	2 vs 3	0.105	0.280
	Group 2		
	4 vs 5	0.296	0.005
	4 vs 6	0.063	0.465
	5 vs 6	0.232	0.0345
Between-group comparison	1 vs 4	−0.042	0.666
	1 vs 5	0.098	0.452
	1 vs 6	0.235	0.0355
	2 vs 5	0.110	0.299
	2 vs 6	0.102	0.250
	3 vs 6	0.510	< 0.001
	1 vs 3	−0.098	0.390
	2 vs 5	0.110	0.299
	3 vs 6	0.510	< 0.001

“Overall” comparison refers to comparison of kappa values of all raters of the both groups

Group 1: Inter-rater group without training (raters 1–3)

Group 2: Inter-rater group with training (raters 4–6)

Between-group comparison shows the comparison between two groups w and w/o training

**Fig. 3:**
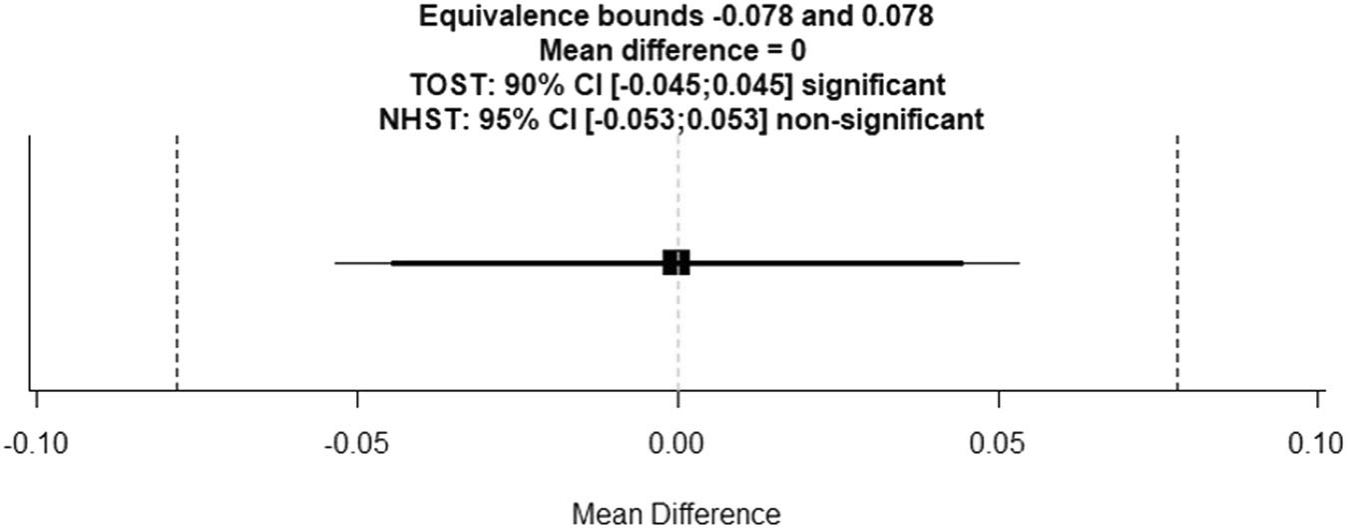
Two one-sided *t*-test graph

Two one-sided *t*-tests were applied between the mean METRICS total score value obtained by readers of groups 1 (without training; 0.63 ± 0.11) and 2 (with training; 0.63 ± 0.11). The lower and upper bounds were calculated to have a statistical power of 0.8 with an alpha of 0.05. Thus, with a lower and upper equivalence bound of ± 0.078 and a mean difference of 0, the *p-*values for both lower and upper bounds were 0.002 (Fig. 3).

**Fig. 4:**
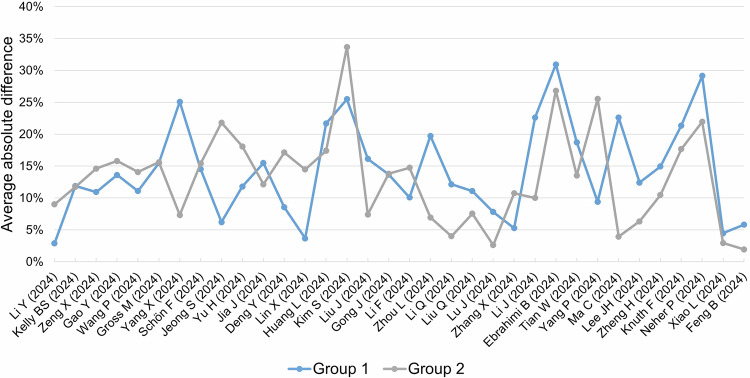
Average of absolute differences in groups 1 (without training) and 2 (with training)

To investigate which papers had very poor reproducibility in inter-rater analysis, we calculated the mean of the absolute difference between total METRICS scores and as a result, the following six papers [27, 35, 36, 46, 52, 53] showed the highest absolute mean difference (> 15%; SD 2.1) in both groups (Fig. 4).

### Intra‑rater reliability

In the intra-rater analysis, the reliability of the METRICS score was truly excellent for two raters (raters 9 and 12), and good to excellent for another two raters (raters 8 and 10). For the remaining two raters, it was poor to good (rater 11) and moderate to excellent (rater 7). Cohen’s k for quality category was moderate to almost perfect, ranging from 0.414 to 1.

Detailed results of intra-rater reliability analysis are shown in Table 5 and Supplementary Material S2.

**Table 5:** Intra-rater analysis for total METRICS score and quality category

Raters’ number	ICC			k (Cohen)	
	Estimate	95% CI	*p*	k	*p*
7	0.857	0.652–0.946	< 0.001	0.590	< 0.001
8	0.958	0.877–0.985	< 0.001	0.700	< 0.001
9	0.979	0.935–0.993	< 0.001	0.890	< 0.001
10	0.960	0.879–0.986	< 0.001	0.800	< 0.001
11	0.736	0.421–0.895	< 0.001	0.414	0.0149
12	0.988	0.968–0.996	< 0.001	1.000	< 0.001

*ICC* intra-class correlation, *CI* confidence interval

### Item-wise analysis

The intra-rater reliability of individual items’ scores was relatively high with most of the items having moderate to excellent intra-rater reliability.

**Fig. 5:**
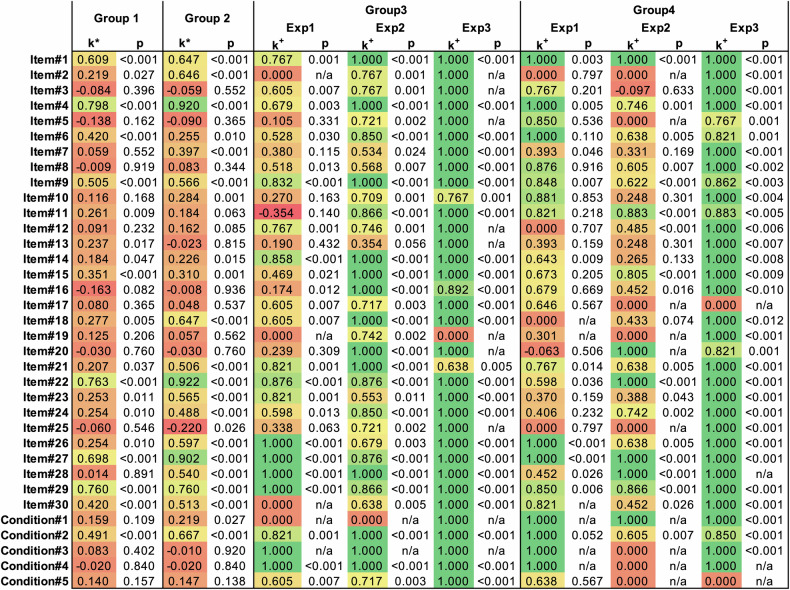
Cohen and Fleiss’ k values of single METRICS items and conditions. Kappa values were not calculated in perfect agreement due to a lack of variance. In this case, kappa values were assumed to be 1. ^*^ Stands for whenever Fleiss’ k was calculated, and ^+^ stands for whenever Cohen’s k was calculated

The inter-rater reliability for METRICS items’ score reproducibility within groups 1 (without training) and 2 (with training) was very low. The only items that had high (substantial to almost perfect) inter-rater agreement were items 1, 4, 22, 27, and 29. Detailed results are summarized in Fig. 5.

#### Post-scoring survey

According to the post-scoring survey that every rater filled out right after they finished their final scoring, we have found out that half of our raters found items #16 and #17 a little challenging to understand and more than half (50% little challenging; 16.7% very hard) of the raters found item #19 little challenging to very difficult. Most raters (75–100%) found the rest of the items very clear and easy to use (Supplementary Material S3).

## Discussion

In this study, we investigated the reproducibility of METRICS to better understand the possible applicability of research papers in different real-world settings. We found that the inter-rater reliability was generally poor to moderate across groups, and this result was not substantially improved by the preliminary training session. This finding is in line with previous analysis on RQS reproducibility where ICC was poor to moderate within groups [13]. Moreover, the highest inter-rater reliability was observed among the most experienced raters (0.869); this finding suggests that experience, intended as a combination of years of practice, prior radiomics study publications, and familiarity with other metrics such as the RQS, might play a role in the accurate application of the METRICS score.

On the other hand, the intra-rater reliability was high in both groups regardless of training. These findings suggest that individual raters can be consistent in their scoring when reassessing the same papers, independent of their training and level of expertise. It should be noted that all readers, even if less experienced and not explicitly trained, were still active members of the EuSoMII Radiomics Auditing Group and interested in the topic of radiomics, which may have contributed to this result.

The analysis of individual METRICS items revealed that, in inter-group comparison, most items exhibited low inter-rater reliability, and only a few items showed moderate to excellent reliability. These results suggest that some components of the METRICS score may be more subjective or difficult to interpret consistently (e.g., items #16, #17, and #19). Nevertheless, the higher intra-rater reliability for these items suggests that single individuals can apply these criteria consistently over time. This result suggests that METRICS reproducibility could benefit from additional explanations and examples to reduce the observed subjectivity, as available for other guidelines and checklists [60–62].

The reproducibility of METRICS quality categories was surprisingly poor in inter-group assessment, except for the comparison between expert raters, where moderate to good agreement was observed. This finding identifies a potential bias in the categorization of the METRICS total score that should be addressed in future investigations and implementations of the score. In intra-rater analysis, the quality category’s reproducibility was higher.

The analysis of papers with the highest absolute mean differences in METRICS scores between raters identified six studies [27, 35, 36, 46, 52, 53] with particularly poor reproducibility (mean absolute difference > 15% in both groups). The cause of this discrepancy could be related to ambiguities in the studies themselves or challenges in applying the METRICS criteria to these specific papers and should be the object of further investigation to improve the applicability of METRICS and its comprehension by operators. This is also an important indication that ambiguities regarding study reporting may lead to lower METRICS score reproducibility when METRICS is used by researchers other than the authors of a study, as authors tend to overestimate their scores when assessing their papers [13]. Therefore, authors are highly encouraged to refer to standardized reporting guidelines, such as CLEAR [63] during the study design and reporting to enhance clarity and consistency. Furthermore, the authors are also encouraged to apply METRICS, calculate their paper’s score, and report it in the final publication for further comparison studies. Nonetheless, such practices should be performed with great care as recent meta-research studies showed that self-reported checklists, including quality scoring tools, frequently include inaccurate and misleading information [64, 65].

After more than a decade since the term radiomics was introduced, METRICS represents a relatively new addition to the scientific toolbox of this field. Nevertheless, it should be noted that investigators have started employing this tool and there are promising results in terms of its reproducibility and ease of use. For example, Aghakhanyan et al reported an ICC of 0.886 between 2 raters using METRICS in pediatric sarcoma radiomics articles [66], while Russo et al reported an ICC of 0.959 across four readers assessing endometrial cancer radiomics studies [67]. These results suggest that, in line with its intended use, METRICS is reliable when employed in systematic quality auditing of radiomics research.

This study has a couple of limitations. First, the selected papers were limited and not necessarily representative of the whole radiomics literature. Second, raters were selected from a small sample of experts in the field, all of them are radiologists or radiology residents, but possibly not fully representative of the rest of the radiomics researchers. Third, raters were categorized based on their experience in radiomics research, but none of them had a consolidated experience in METRICS assessment. Fourth, although we did not find any positive effects of training on reproducibility, we must acknowledge that a single training session could not be enough to give operators sufficient experience in METRICS assessment. Fifth, some raters were involved in the development of the original METRICS initiative. This prior involvement might have led to a shared baseline understanding or subtle biases that could influence the ratings, even though this influence appears minimal, based on the results of this study. Finally, the intra-rater group evaluated a subset of papers after a washout period. While the total number of papers assessed across both groups amounted to 34, it would have been more appropriate to ensure both groups evaluated the exact same set of 34 papers for consistency. Nevertheless, our previous paper on RQS had the same design, and only by using the same methodology we were able to achieve a more effective comparison between RQS and METRICS reproducibility.

In conclusion, our findings show a relatively low reproducibility of METRICS score between different raters and  relatively high reproducibility within raters. This highlights the need for further efforts to achieve a common understanding of METRICS items. In this context, the development of resources like the Explanation and Elaboration with Examples for CLEAR (CLEAR-E3) [60] appears to be crucial for improving the reproducibility and we will soon extend our efforts to METRICS and prepare an Explanation and Elaboration with Examples paper also for METRICS to enhance its usability and robustness. We hope the current study provides a foundation for future efforts aimed at improving METRICS, particularly with its publication and item-level detailed analyses.

## Supplementary information

Supplement S1_group 1_and_group_2_rawdata

Supplement S2_group 3_and_group_4_rawdata

Supplement S3

## Abbreviations

EuSoMII: European Society of Medical Imaging Informatics
ICC: Intraclass correlation coefficient
k: Kappa statistics
METRICS: Methodological radiomics score
Q1: First quartile
Q2: Second quartile
RQS: Radiomics quality score
TOST: Two one-sided *t*-tests
